# An Appraisal of the Current Scenario in Vaccine Research for COVID-19

**DOI:** 10.3390/v13071397

**Published:** 2021-07-18

**Authors:** Wai Chin Chong, Dinesh K. Chellappan, Shakti D. Shukla, Gregory M. Peterson, Rahul P. Patel, Niraj Kumar Jha, Rajaraman D. Eri, Kamal Dua, Murtaza M. Tambuwala, Madhur D. Shastri

**Affiliations:** 1 Department of Molecular and Translational Science, Monash University, Clayton 3168, Australia; waichin.chong@hudson.org.au; 2 Department of Life Sciences, School of Pharmacy, International Medical University (IMU), Kuala Lumpur 57000, Malaysia; Dinesh_Kumar@imu.edu.my; 3 Discipline of Pharmacy, Graduate School of Health, University of Technology Sydney, Sydney 2007, Australia; shakti.shukla@newcastle.edu.au (S.D.S.); Kamal.Dua@uts.edu.au (K.D.); 4 School of Pharmacy and Pharmacology, University of Tasmania, Hobart 7005, Australia; g.peterson@utas.edu.au (G.M.P.); Rahul.Patel@utas.edu.au (R.P.P.); 5 Department of Biotechnology, School of Engineering & Technology (SET), Sharda University, Greater Noida 201310, UP, India; nirajkumarjha2011@gmail.com; 6 School of Health Sciences, College of Health and Medicine, University of Tasmania, Launceston 7248, Australia; rajaraman.eri@utas.edu.au; 7 School of Pharmacy and Pharmaceutical Science, Ulster University, Coleraine BT52 1SA, UK

**Keywords:** COVID-19, SARS-CoV-2, vaccines, therapeutic targets, immunity

## Abstract

The recent coronavirus disease 2019 (COVID-19) outbreak has drawn global attention, affecting millions, disrupting economies and healthcare modalities. With its high infection rate, COVID-19 has caused a colossal health crisis worldwide. While information on the comprehensive nature of this infectious agent, SARS-CoV-2, still remains obscure, ongoing genomic studies have been successful in identifying its genomic sequence and the presenting antigen. These may serve as promising, potential therapeutic targets in the effective management of COVID-19. In an attempt to establish herd immunity, massive efforts have been directed and driven toward developing vaccines against the SARS-CoV-2 pathogen. This review, in this direction, is aimed at providing the current scenario and future perspectives in the development of vaccines against SARS-CoV-2.

## 1. Introduction

The current pandemic, coronavirus disease 2019 (COVID-19), has officially been confirmed in more than 187 million individuals and caused in excess of four million deaths globally (as of 12 July 2021) [1,2]. The causative pathogen, severe acute respiratory syndrome (SARS)-coronavirus 2 (SARS-CoV2) belongs to the coronavirus family, which is a large family of enveloped viruses that contain a positive sense, single-stranded RNA genome [3]. In the recent past, several members of the coronavirus family have been involved in significant disease outbreaks globally. The major diseases in this category include SARS and Middle East respiratory syndrome (MERS). In late 2019, SARS-CoV-2 was first detected in people who had visited the wet markets in the city of Wuhan, China. The viral particles soon engulfed major parts of the globe in a chaotic manner, resulting in an uncontrollable pandemic outbreak. A number of studies have reported that SARS-CoV-2 attacks the host’s respiratory system and thereby induces pneumonia-like syndrome, which may even lead to death in severe conditions [4]. To date, there are no effective and proven medications available to treat the disease in a synchronized manner, although a number of drugs and antiviral agents are being administered in an effort to contain the viral spread and alleviate the inflammatory response in patients presenting with severe complications.

Simultaneous efforts, to considerable success, are being made to anticipate the severity and transmission potential of SARS-CoV-2 and to develop efficacious prophylactic approaches, including public health messaging/advisories (lockdowns/hygiene/social distancing) and, more importantly, the development of vaccines. To date, a number of candidate vaccines are under various stages of development. A total of 8 of them have been approved for use, 121 are in clinical development, and 184 are in the preclinical development phase [5]. In this rapid review, we summarize the utility of vaccines to contain COVID-19 in the longer term and elaborate on major vaccine candidates currently in use in various countries.

## 2. Vaccination as a Promising Strategy Against COVID-19

Vaccination is a type of immunotherapy that introduces an immunogenic material to artificially stimulate the adaptive immune response of the body. Its applications and efficacy in combating and eradicating deadly infectious diseases such as smallpox, poliomyelitis, human papillomavirus, and several other infectious diseases have been well-documented [6,7,8,9]. Hence, it is by far the primary strategy in contesting the COVID-19 pandemic [10]. Findings from the recent next-generation sequencing studies have revealed approximately 5500 full-length genomes of SARS-CoV-2 that were isolated from various countries. These findings facilitate delineating the polymorphisms in the S protein and other important proteins of the virus that may serve as potential targets in the development of vaccines [11,12,13,14]. Interestingly, there is little to no cross-neutralization between the SARS-CoV and SARS-CoV-2, proposing that recovery from one infection may not necessarily provide any immune protection against the other [15]. Hence, there is a compelling need for a new vaccine agent that could exclusively target the SARS-CoV-2 pathogen. Notably, the development is highly possible with the reference and aid of the available next-generation sequencing database.

Besides the vaccine target, the production platform also plays an important role in determining the efficacy of the product in question. There are multiple distinct vaccine platforms that are currently available. These may include live-attenuated virus vaccine, inactivated virus vaccine, protein subunit vaccine, viral vector transduction-based vaccine, and nucleic acid-based vaccine, to name a few. Each platform has its own distinct advantages and disadvantages [16]. The classical vaccine platforms are the live-attenuated viral vaccine, the inactivated virus vaccine, and the protein subunit vaccine [16]. There exist a series of vaccines containing viral antigen, either live-attenuated virus, inactivated virus, synthetic or recombinant antigenic protein, that serve as immunogenic antigens to trigger a strong and long-lasting adaptive immune response. Live-attenuated vaccines typically use a living target virus, which had been weakened and no longer possesses the ability to infect, to stimulate the body’s immune system to elicit an adaptive immune response against the target virus [17]. As compared to an inactive virus or protein subunit vaccine, the live-attenuated vaccine is an exemplar vaccine model that mimics the actual infectious process of the infectious agent without causing a pathogenic infection [17,18]. However, the disadvantage of this vaccine platform is, it may not be suitable to the population who have weak or compromised immune systems, including those with long-term health conditions, the elderly, or who had organ transplantation performed before vaccination [19,20,21,22]. For these specific groups of the population, inactivated virus or protein subunit vaccine may be more suitable as vaccine candidates. Inactivated virus vaccine uses dead virus as the immunogenic antigen, whereas a protein subunit vaccine uses a fragment of the virus protein as the antigen to trigger an immune response [18,23,24]. As for COVID-19, the spike protein of the SARS-CoV-2 is the most suitable target, as it holds an essential role in regulating the binding of the virus with the host cells [15,25,26]. As compared to live-attenuated vaccines, inactivated virus and protein subunit vaccines do not essentially contain any living virus; hence, they are considered relatively safe [18,24]. However, the classical vaccine platform, including both types of the above-described vaccines, possess relatively lower immunogenicity and require additional adjuvants or boosters to enhance their biological half-lives and potentiate elicited immune responses [17,18,23,24,27].

With the emerging challenges and increasing drawbacks associated with the current vaccine development platforms, the global attention has now turned toward next-generation vaccine platforms, which essentially consist of, viral vector-based and nucleic acid-based vaccine platforms [16]. In particular, the viral vector transduction-based vaccine has been demonstrated to be a promising vaccine platform. Unlike the conventional vaccine, this type of vaccine uses host cells to produce target immunogenic antigens for adaptive immune response by viral transduction of the genetic code into host cells via modified viral vectors [28]. Viral vector transduction-based vaccines may be further differentiated into replicating or non-replicating types. A replicating vaccine may infect host cells and transduce gene sequences for both viral vectors and the target antigen to produce more infectious viral vectors that are able to infect more host cells; whereas a non-replicating vaccine only transduces gene sequences for the target antigen, hence, restrict self-replication of the viral vector [29,30,31]. As there is no living infectious agent that is involved during the vaccination process, this vaccine platform is relatively safe, and furthermore, prevents the need of handling the infectious agent [32]. Furthermore, this vaccine platform offers a long-term antigenic immune response against the target pathogens, as the genetic code for the immunogenic antigen of the pathogen is constitutively expressed in host cells after the vaccination [32]. However, the vaccine has relatively low efficiency, as the host may react against the viral vector during the first exposure and produce an unspecific immune response [30,31].

Meanwhile, the nucleic acid-based vaccine may be further classified as DNA or mRNA-based vaccines [16]. A DNA-based vaccine is a type of vaccine that consists of a synthetic DNA plasmid-construct encoding the target virus’s antigen [33]. Unlike the conventional vaccine administration, a DNA-based vaccine requires additional electroporation after the vaccine administration to facilitate the uptake of DNA-construct into the host cell [33]. On the other hand, mRNA-based vaccines use a similar principle, except in, they bypass the nuclear translocation and mRNA transcription process [34,35]. As both mRNA and DNA vaccines do not use any extracted viral material, the vaccination process is safe and is suitable for most of the population [35,36,37]. As synthetic DNA is temperature stable, it poses significant advantages from the point of view of mass production, delivery, and storage [36]. As compared to DNA, mRNA-based vaccines are relatively unstable and are temperature-sensitive, hence require extensive precautions in the developing and handling process [36,37]. Nevertheless, as both vaccine platforms are relatively new, there is an insufficient number of studies to determine their possible adverse effects on the receivers.

## 3. COVID-19 Vaccine Development

To further fast-track the development of counter-measures against the COVID-19 pandemic, the WHO has declared the outbreak as a Public Health Emergency of International Concern, as well as has initiated a Research and Development Blueprint under the recommendation of the WHO emergency committee [38]. The blueprint aims to “accelerate innovative research to help contain the spread of the epidemic and facilitate care for those affected”. It further emphasizes to “support research priorities that contribute to global research platforms in hopes of learning from the current pandemic response to better prepare for the next unforeseen epidemic” [38]. Since then, massive research funding and government support have been channeled toward the development of vaccines. In accordance with the blueprint, currently, there are more than 40 vaccine candidates that are prepared to progress until the clinical trial phase, as of 29 June 2021, as shown in Table 1.

### 3.1. BNT162b2

BNT162 is a series of mRNA-based vaccine candidates developed by Pfizer and BioNTech as a measure to contain COVID-19 spread. These vaccines are essentially lipid nanoparticles containing mRNA that encodes the SARS-CoV2 antigens and expresses 1 of 2 antigens for the SARS-CoV-2 full-length, P2 mutant, prefusion spike glycoprotein (P2 S) (version 9) (Genbank: MN908947); or a trimerized SARS-CoV-2 spike glycoprotein receptor-binding domain (RBD) (version 5) [39]. Notably, vaccine candidates, BNT162b1 (variantRBP020.3; nucleoside-modified messenger RNA (modRNA) with blunted innate immune sensor-activating capacity and augmented expression encoding the RBD) and BNT162b2 (variant RBP020.2; nucleoside-modified messenger RNA (modRNA) as above, but encoding P2S) have demonstrated their efficacy in vivo by “inducing protective antiviral effects rhesus macaques, with concomitant high neutralizing antibody titers and a TH1-biased cellular response in rhesus macaques and mice” [39,40]. Under a phase 3 clinical trial setting (NCT04368728) with 195 healthy adult participants, both BNT162b1 and BNT162b2 demonstrated similar immunogenicity [41]. However, the trial also discovered that participants who received the BNT162b1 vaccine suffered greater adverse effects as compared to those individuals who received the BNT162b2 variant, where the adverse effects ranged from mild fever (38–40 °C) to moderate systemic effects such as fatigue, headache, and chills. The observations and findings have led to rising concerns on the safety and tolerability of BNT162b1 [41]. Similarly, another clinical trial study with 60 healthy adult participants found that participants who received two doses between 1 and 50 µg of BNT162b1 had robust RBD-specific antibody, T cell, and favorable cytokine responses [42]. However, due to the relatively small population size, it is still obscure if the adverse effects exclusively occur only in a certain population who received BNT162b1. BNT162b2 was authorized by the Medicines and Healthcare products Regulatory Agency (MHRA) for use in the U.K. on 2 December 2020 after a rolling review of vaccine data submitted by Pfizer and BioNTech, despite the vaccine candidate having not completed its planned clinical trials assessment [43]. Following the approval, the United States of America Food and Drug Administration (USFDA) released their independent analysis of the clinical trial conducted on the vaccine candidate, in which they discovered the candidate has about 95% vaccination efficacy without eliciting serious adverse events [44]. Similarly, a recent clinical study in Israel also reported the high efficacy of BNT162b2 in protecting the receiver against COVID-19, with a vaccine efficacy of around 90% [45]. The observations and findings have further attracted global attention as it was one of the notable vaccine candidates against COVID-19, leading WHO to list BNT162b2 in Emergency Use Listing for COVID-19 [46]. So far, multiple countries have approved BNT162b2 as a COVID-19 vaccine, not limited to Argentina, Canada, Chile, Costa Rica, Ecuador, Jordan, Kuwait, Mexico, Panama, Singapore, Bahrain, Saudi Arabia, and Switzerland [47,48,49,50,51,52,53,54,55,56,57]. Notably, a recent observational study with a total number of 9876 participants who received a complete dosage of BNT162b2 (4938 vaccinated, 4938 unvaccinated) demonstrated a significant reduction in SARS-CoV-2 viral load (*p* < 1 × 10^−17^), further pointing toward the efficacy of this vaccine against SARS-CoV-2 [58]. So far, the BNT162b2 vaccine has passed phase 1, 2, and 3 clinical trials and currently undergoing a phase 4 clinical trial (NCT04760132) with 10,000 participants [59].

### 3.2. Sputnik V

Sputnik V, also known as Gam-COVID-Vac, is a non-replicating viral vector vaccine developed by the Gameleva Research Institute in collaboration with the Russian Direct Investment Fund and The Gamaleya National Research Center for Epidemiology and Microbiology of the Ministry of Health of the Russian Federation. It consists of a recombinant adenovirus type 26 (rAd26) vector and a recombinant adenovirus type 5 (rAd5) vector, in which both encoded the SARS-CoV-2’s spike glycoprotein (rAd26-S and rAd5-S). By using a replication-competent EGFP-reporter vesicular stomatitis virus system, it consists of recombinant DNA, rcVSV-CoV2-S, which encodes spike glycoprotein from SARS-CoV-2 (Genbank: MN908947.3), and packed in recombinant adenovirus type 26 (rAd26) vector and a recombinant adenovirus type 5 (rAd5) vector [60].

Unlike other vaccine candidates, the Sputnik V trial protocol has not been made public. Moreover, there are limited preclinical and clinical trial data available with regards to the Sputnik V vaccine. So far, there are a total of 2 phase 1/2 clinical trial studies (NCT04436471 and NCT04437875) that were conducted with a total of 76 healthy adult participants. The preliminary observations on the vaccine trial have been published [60]. According to both the concluded trials, participants has developed antibodies against SARS-CoV-2 glycoprotein after being administered with the Sputnik V vaccine, without any serious adverse events [60]. Currently, a phase 2 trial of 110 participants who are older than 60 years (NCT04587219) and a phase 3 trial of about 40,000 participants at multiple centers in Russia (NCT04530396) are being conducted for the Sputnik V vaccine to further determine its safety and efficacy profile under larger population trial [61,62]. Despite having a smaller pool of tested participants and limited clinical trial data, the Sputnik V vaccine was quickly approved and was registered on 11 August 2020 by Russian Federation [63]. This approval has drawn strong criticisms from various quarters from among the international scientific community for lack of data on safety and efficacy and for not following proper clinical trial safety guidelines [64,65]. So far, the vaccine candidate was granted approval in various countries, including Belarus, Argentina, Algeria, Bolivia, Serbia, and Palestine [66,67,68,69,70,71].

### 3.3. EpiVacCorona

EpiVacCorona is a peptide vaccine developed by the Russian Federal Budgetary Research Institution, the State Research Centre of Virology and Biotechnology. It consists of 3 distinct amino acids: (1) CRLFRKSNLKPFERDISTEIYQAGS, (2) CKEIDRLNEVAKNLNESLIDLQE, and (3) CKNLNESLIDLQELGKYEQYIK, which are artificially synthesized small fragment-peptide antigens of SARS-CoV-2 protein, conjugated to a carrier protein and adsorbed on an aluminum-containing adjuvant (aluminum hydroxide) [72]. Its phase 1/2 clinical trial (NCT04527575) was reported with excellent efficacy, with a 100% response rate and seroconversion with a neutralizing antibody titer >1:20, 21 days following complete vaccine administration. Moreover, all participants are reported free from any severe local or systemic adverse events [72,73]. Currently, it is under a phase 3 clinical trial targeted at 3000 participants [74]. EpiVacCorona was granted emergency approval from the Russian government on 15 October 2020 [75,76].

### 3.4. CoronaVac (PiCoVacc)

CoronaVac is a formalin-inactivated, alum-adjuvanted vaccine developed by the Sinovac Biotech Company. It is developed from purified inactivated SARS-CoV-2 virus strain CN2, which was isolated from the bronchoalveolar lavage fluid from one of the 11 hospitalized patients infected with SARS-CoV-2 [77]. The CN2 strain is selected as it was closely related to SARS-CoV-2 strain 2019-nCoV-BetaCoV Wuhan/WIV04/2019 (GISAID accession ID = EPI_ISL_402124), which has been firstly reported as the main source of the infectious agent for the coronavirus pandemic [77,78,79]. Under preclinical setting, CoronaVac vaccine demonstrated distinct safety and immunogenicity profile in various animal models, including Wistar rat and rhesus macaque monkey models, in which the animals survived and produced a high titer of neutralizing antibodies without developing serious adverse events [77]. Furthermore, a recent phase 1/2 clinical trial (NCT04352608) with 743 healthy participants demonstrated similar outcomes, with positive seroconversion in most participants (92.4% of participants after being administered with a 3 μg dose, on a 0–14 day schedule and 97.4% after being administered with a 3 μg the same dose on a 0–28 day schedule) [80]. Notably, another safety and tolerability trial targeting the elderly population (aged 65–68) (NCT04383574) further showed that the participants who received a complete dosage of CoronaVac did not show severe adverse reactions, and about 97% of participants showed at least four-fold neutralizing antibody titer, suggestive of the vaccine candidate well tolerable and effective among the elderly community [81]. Due to its distinct safety and immunogenicity efficacy profile, the Government of China has fast-tracked the production and has given emergency approval for the administration of CoronaVac exclusively to high-risk essential populations, such as health care workers, doctors, and nurses [82]. Currently, several international phase 3 clinical trials are in progress for the CoronaVac in Brazil (NCT04456595), Turkey (NCT04582344), and Indonesia (NCT04508075) [83,84,85].

### 3.5. mRNA-1273

mRNA-1273 is a vaccine candidate developed by Moderna Inc. in collaboration with the Coalition of Epidemic Preparedness Innovations and the National Institute of Allergy and Infectious Disease. It is a novel lipid nanoparticle-encapsulated mRNA-based vaccine developed against SARS-CoV-2. The mechanism is based on the principle that cells intake non-replicating mRNA, translate, and transiently express viral antigen protein on the cell surface without entering the cellular nucleus or interacting with the genome constitutively [86]. mRNA-1273 encodes for the full-length spike protein of SARS-CoV-2 (Genbank: MN908947.3), which gets further modified to introduce two proline residues that stabilize the spike protein in a prefusion conformation [86]. Several studies have shown that the spike protein regulates adherent and admission of the virus into host cells upon infection, hence presented as a potential target for the infection [87,88,89,90]. Under in vivo setting, the mouse models administered with the mRNA-1273 vaccine showed a reduction in viral replication and induced a potent neutralizing antibody against SARS-CoV-2 virus, as well as, CD8^+^T cell response. This mechanism has provided immune protection against SARS-CoV-2 infection in the lungs and noses of the mice, without evidence of immunopathology [91]. Similar events were also observed in non-human primate models, in which those vaccinated with the mRNA-1273 triggered a robust immune response against SARS-CoV-2 upon a challenge with the infection [92]. Meanwhile, under a clinical setting, a phase 1 trial demonstrated the efficacy of the vaccine candidate in upregulating SARS-CoV-2 neutralizing antibody titer in 45 healthy adult participants (aged 18–55 years old) and 40 healthy elderly (aged > 56 years old) [93,94]. In the cohort, three participants reported severe local adverse reactions (erythema and induration), and another three participants reported severe systemic adverse reactions (fever, fatigue, nausea, and myalgia) [93,94]. Nonetheless, all adverse reactions resolved within 24 h, proposing that the vaccine candidate is well tolerated by the healthy adult and elderly [38,95]. Meanwhile, in phase 3 efficacy, safety, and immunogenicity trial (NCT04470427) with 30,420 participants (15,210 participants each in vaccine receivers and placebo control group) demonstrated 94.1% of vaccine efficacy in preventing COVID-19 illness after receiving two complete dosages of vaccine, without having any severe adverse side effects [96,97,98]. This study supports that mRNA-1273 is safe to be used as an efficacious vaccine against COVID-19. On 30 November 2020, Moderna requested an emergency use authorization from the FDA and additionally a conditional marketing authorization from the Europen Medicines Agency (EMA) [99,100]. In the meantime, U.K.’s MHRA and Switzerland’s Swissmedic regulator initiated a review on mRNA-1273, which will allow a quick approval process for the vaccine candidate [101,102]. On 19 December 2020, FDA authorized mRNA-1273 for emergency use in the United States of America [103]. Following that, Canada, European Union, Israel, Switzerland, and United Kingdom approved the usage of mRNA-1273 [104,105,106,107,108].

### 3.6. AZD1222

AZD1222 (also known as Oxford-AstraZeneca vaccine, ChAdOx1 nCoV-19) is a COVID-19 vaccine candidate developed by the Oxford University and AstraZeneca. It is a non-replicating primate adenovirus vector containing the sequence of SARS-CoV-2 S protein (Genbank: MN908947) and tissue plasminogen activator. Its preclinical study with mice (BALB/c and CD1) and primate (rhesus macaque) models demonstrated its efficacy in eliciting an immune response against the SARS-CoV-2, including the inducing of viral-specific antibodies and the activation of T-helper 1 immune response (high level of IFN and TNF; low level of IL-4 and IL-10) post-vaccination [109]. Moreover, under a clinical setting, a phase 1/2 trial demonstrated the efficacy of the vaccine candidate in inducing an immune response against SARS-CoV-2 after vaccination [110]. In addition, all participants were found to be well tolerated to the vaccine, and no serious adverse events were reported [110]. However, during its phase 2/3 trial, several adverse cases were reported, such as an unexplained neurological symptom developed by one of the participants in the United Kingdom and a volunteer who died during the trial in Brazil, which forced the trial to be suspended for safety review [111,112]. On 23 October 2020, FDA approved the trial to be restarted after the trial passed its safety review [113]. The preliminary findings of the phase 2/3 trial were published, and it demonstrated 90% vaccination efficacy in 420 healthy adult volunteers without developing any significant adverse events [114]. However, the result raised concern as some participants involved were administered half the actual required dosage. AstraZeneca later admitted that it was a mistake [115]. Despite this, the vaccine candidate was granted emergency authorization by MHRA, Argentina’s National Administration of Drugs, Food, and Medical Devices (ANMT), and Mexico’s Health Regulator “Comision Federal para la Proteccion contra Riesgos Sanitarios” (COFEPRIS) [116,117,118]. On 1 January 2021, India approved the vaccine candidate, with the condition that the vaccine will be manufactured by the Serum Institute of India and will be released as “Covishield” vaccine [119]. Recently, a vaccine efficacy result for AZD1222 with 11,636 participants was reported with an overall vaccine efficacy of 90.0% (67.4%–97%) for those who received two standard doses and 70.4% for those who received one standard dose [120]. Notably, several patients were reported with prothrombotic immune thrombocytopenia after receiving AZD1222 [121,122,123,124]. Patients involved are presented with a thrombotic disorder, including cerebral venous sinus thrombosis, splanchnic vein thrombosis, arterial cerebral thromboembolism, or thrombotic microangiopathy [121,122,123,124]. Furthermore, the hematologic analysis found the presence of anti-platelet factor 4 autoantibodies in patients’ sera, which targets healthy donor platelet in an AZD1222 dependent manner, despite no previous history of heparin-induced thrombocytopenia [121,122,123,124]. Importantly, such aggregation can be resolved and suppressed by heparin [121,122,123,124]. Collectively, AZD1222 demonstrated vaccine efficacy against COVID-19, but its application is associated with severe autoimmune prothrombotic disorders.

### 3.7. Covaxin (BBV152)

Covaxin (also known as BBV-152) is a COVID-19 vaccine candidate developed by the Bharat Biotech and the Indian Council of Medical Research (ICMR) [125]. It is an inactivated vaccine that consists of the whole-virion SARS-CoV-2 inactivated via the β-propiolactone inactivation method [126]. Under preclinical setting, Covaxin demonstrated remarkable immunogenicity and protective efficacy against SARS-CoV-2 in both hamster and rhesus macaques models [126,127]. Within the hamster model, the vaccinated group developed an immune response against the SARS-CoV-2 virus with an increased titer of neutralizing antibodies and rapid clearance of the virus from the lower respiratory tract, reduced virus load in the upper respiratory tract, and absence of lung pathology after vaccination [126]. Similar events also were observed in rhesus macaques models [127]. On the other hand, under a clinical setting, a phase 1 trial with 375 healthy adult participants demonstrated the production of increased neutralizing antibodies against the SARS-CoV-2 virus without developing any serious adverse events [128]. The findings were further supported by its phase 2 trial that was conducted with 380 participants, where seroconversion rates of neutralizing antibodies reached up to 73% without causing any serious adverse events after vaccination [129]. On 3 January 2021, the Indian government approved Covaxin as a COVID-19 vaccine in India [130]. Such quick approval drew global criticism within the international scientific community for lack of data on safety and efficacy and for not following standard clinical trial safety guidelines [131,132,133].

### 3.8. Ad26.COV2

Ad26.COV2 (also known as Janssen COVID-19 vaccine or Johnson & Johnson COVID-19 vaccine) is a vaccine candidate developed by Janssen Pharmaceutical in collaboration with Johnson & Johnson pharmaceutical company [134]. It is a recombinant adenovirus serotype 26 vector encoding SARS-CoV-2 peplomers based on SARS-CoV-2 Wuhan-Hu-1 isolate strain (Genbank accession number: MN908947) [135]. Ad26.COV2 demonstrated high efficacy under preclinical setting in multiple animal models, including Syrian hamster, non-human primates, in which all vaccinated animals showed a rapid increase in neutralizing antibodies and reduction in lung viral load [136,137,138,139]. Notably, all animal models reported the absence of any adverse side effects or signs of vaccine-related respiratory disease, suggestive of a safe and tolerable vaccine candidate against SARS-CoV-2 [136,137,138,139]. Under clinical setting, safety and tolerability trial (NCT04436276) exhibited high safety and efficacy profile of Ad26.COV2 in human participants, with elevated neutralizing antibody response of 88% in the adult population (aged 18–55) and 93% in the elderly population (aged > 65) without any significant adverse events reported [140]. On the other hand, phase 3 trial (NCT04505722) with 43,783 (21,895 received vaccine; 21,888 received placebo) participants revealed vaccine efficacy of approximate 66.8% [141]. Moreover, there are only 18 participants who reported pyrexia, which is resolved by using analgesics or antipyretics 7 days after vaccination [141]. Due to the excellent efficacy, FDA issued an emergency use authorization for Ad26.COV2 for the prevention of COVID-19 on 27 February 2021 [142], followed by the conditional marketing authorization by EMA [143] and MHRA [144]. However, increasing case reports found that some patients who received the Ad26.COV2 vaccine develop thrombotic thrombocytopenia symptoms [145,146,147]. In response, the manufacturers acknowledge the presence of rare thrombotic thrombocytopenia among the vaccine receiver but argue that the reporting rate is less than 1:1,000,000, and more evidence and studies are needed to clarify such observations [146].

### 3.9. BBIBP-CorV

BBIBP-CorV is an inactivated viral vaccine manufactured by Sinopharm pharmaceutical company in collaboration with the Chinese Center for Disease Control and Prevention and the Beijing Institute of Biological Products. It is an aluminum hydroxide-based adjuvant, β-propiolactone-inactivated vaccine according to the 19nCOV-CDC-TAN-HB02 strain (HB02 strain) [148]. Under a preclinical setting, the BBIBO-CorV vaccine was able to induce active seroconversion, leading the host to develop a significantly high titer of neutralizing antibodies in immune-competent mice models [149]. Similar positive effects were also demonstrated in other non-human animal models, including rabbit, guinea pig, rat, and rhesus macaques models [149]. Furthermore, it also demonstrated a suitable safety profile, in which the animal models that received the vaccine did not show any sign of ethical endpoint or ill-health after vaccination, suggestive of the safe and efficacious vaccine candidate. On the other hand, under the clinical trial setting, its safety and tolerability trial (ChiCTR2000032459) demonstrated that participants who received the vaccine have significantly elevated neutralizing antibody titer levels in their sera; 14 days after vaccination, with the seroconversion rates of approximately 100% [150]. Furthermore, no patients were reported with severe adverse events within 28 days after vaccination [150]. Whereas, in immunogenicity trial (NCT04510207) with 45,000 healthy participants, the vaccine candidate demonstrated excellent efficacy in preventing against COVID-19, with the seroconversion of neutralizing antibody of approximate 99.5% among adult participants (aged 18–59) and 100% among elderly participants (aged > 65) [150]. In a preliminary analysis based on the available data, BBIBP-CorV has an efficacy of 78.1% in protecting against COVID-19 [150]. Similarly, no severe adverse events were reported in participants who received the vaccination, suggesting that BBIBP-CorV is safe and tolerable in healthy participants [150]. Due to its high safety and efficacy profile, WHO granted approval for it to be used in the COVID-19 Vaccine Global Access program, and it is currently being used by multiple nations [151,152].

## 4. Efficacy of Vaccine Against Emerging SARS-CoV-2 Variant Strains

**Figure 1:**
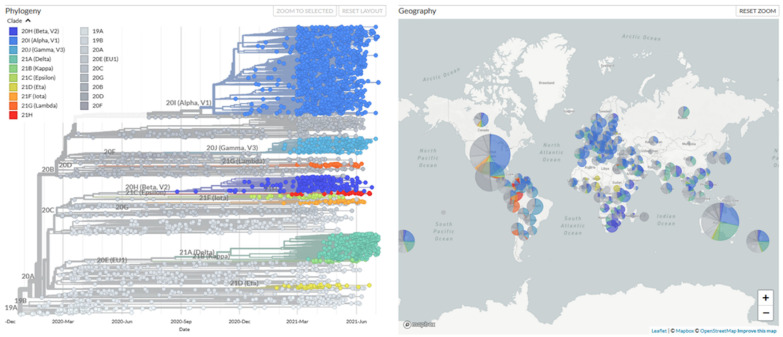
The phylogeny tree of SARS-CoV-2 variant strains. The figure depicts all discovered variant strains obtained from 3916 genome samples between December 2019 till July 2021 and their prevalence across the globe. The real-time data to prepare this phylogeny tree was obtained from [167].

Increasing case studies have reported new variants of SARS-CoV-2 virus strains diagnosed in people around the world. By definition, a SARS-CoV-2 variant is a mutated strain of SARS-CoV-2, in which the mutated strain may possess different replication, transmission, or virulence rate as compared to its parental strain [153,154,155,156,157]. Notably, the mutated strain was reported with enhanced resistance or immune escape mechanisms against current vaccine candidates; hence, the vaccine developed so far may lose partial or complete efficacy in combating these mutated variant strains, posing an extreme challenge in combating the COVID-19 pandemic [157,158,159,160,161,162]. The earliest variant discovered is known as the SARS-CoV-2 Alpha variant (B.1.1.7), which was found and documented in United Kingdom [163]. Since then, multiple variant strains, such as SARS-CoV-2 Beta variant (B.1.351, South Africa) [164], Gamma variant (P.1, Brazil) [165], or Iota variant (B.1.526, United States of America) [166] were reported across the countries. So far, there are at least 19 lineage variants of SARS-CoV-2 were discovered and documented, as summarized in Figure 1 [167].

Fortunately, some vaccine candidates were found to be efficacious against these variants. For instance, the BNT162b2 vaccine had an estimated vaccination efficacy of 75% against the Beta variant, 88% against the Delta variant (B.1.617.2), and 93% against the Alpha variant of SARS-CoV-2 [168,169]. BBIBP-CoV showed that patients who were diagnosed with variant strain GDPCC (501Y.V2) and BJ01 (D614G) produced a high titer value of neutralizing antibody after receiving BBIBP-CoV vaccine, although the neutralization titer is relatively lower than compared to its target strain (HB02 = 110.9 vs. BJ01 = 107.2 vs. GDPCC = 70.9), suggesting that the vaccine candidate provided certain protections against the variant strains [170]. On the other hand, the mRNA-1273 demonstrated a reduction in seroconversion rates and neutralizing antibody titer level against the Beta, Gamma, and Alpha variants, concerning its effectiveness in protecting against COVID-19 variant strains [171]. Collectively, these findings highlight the limited efficacy of the current vaccine candidates against the SARS-CoV-2 variant strains, urging for a need to develop a better vaccine candidate with higher vaccine efficacy against these variants.

## 5. Conclusions

In this review, we have appraised the current state of the COVID-19 pandemic and the vaccines that have been approved so far in the fight against this viral disease. However, as SARS-CoV-2 is a novel organism that was recently identified, the information regarding this infectious agent remains obscure. Nonetheless, recent genome sequencing data may shed more light with regards to the pathogenesis of SARS-CoV-2, which will prove useful in the development of effective treatments against it. On the other hand, thanks to the accelerated track in vaccine development, which has resulted in the development and approval of several COVID-19 vaccines to date. Furthermore, due to the large number of trials that are in progress currently, there will be more vaccines to be approved in the near future. Notably, due to the emergence of new SARS-CoV-2 variant strains, vaccine candidates showing effectiveness on all variants are urgently needed.

## 6. Opinion

The situation of the COVID-19 pandemic is constantly changing, and there are constant updates regarding SARS-CoV-2 and its disease pathology, as well as the information on vaccines developed against the virus. The cases of COVID-19 are constantly on the rise in several countries, which has become unavoidable. If unchecked, the global viral infectivity rates may prove catastrophic. This has led to the urgent need to fully vaccinate society against the COVID-19 pathogen. Currently, the available vaccines are under slow progression for mass immunization, as the supply has been largely limited. Therefore, effective measures and suitable interventions are needed to further accelerate the vaccine development to eradicate the COVID-19 infection.

## 7. Key Findings

- SARS-CoV-2 has been the causative viral pathogen responsible for the COVID-19 outbreak;
- Vaccination against SARS-CoV-2 has now become the main therapeutic strategy for eradicating COVID-19 from the community;
- Numerous vaccine candidates have been introduced, of which mRNA 1273, BNT162, AZD1222, CoronaVac, Sputnik V, EpiVaCorona, and Covaxin have been the leading vaccine candidates against SARS-CoV-2.

## Figures and Tables

**Table 1:** Current COVID-19 vaccine candidates.

Vaccine Type	Candidate Vaccine	Developers	Trial Phase	Number of Virologically Confirmed Symptomatic Cases of COVID-19	Efficacy Compared to Placebo for the Prevention of SARS-CoV-2	Efficacy of Vaccine Against Severe and non-Severe COVID-19	Efficacy: Seroconversion Rates	Assess Humoral Immunogenicity	Safety and Immunogenicity of a Booster Dose
Inactivated virus	CoronaVac	Sinovac Research and Development Co., Ltd	4	X	X	X	X	X	X
Inactivated virus	Vero cell	Sinopharm + China National Biotec Group Co + Wuhan Institute of Biological Products	3	X	X		X	X	X
Inactivated virus	BBIBP-CorV	Sinopharm + China National Biotec Group Co + Beijing Institute of Biological Products	4	X	X	X	X	X	X
Viral vector (Non-replicating)	AZD1222	AstraZeneca + University of Oxford	4	X		X	X	X	X
Viral vector (Non-replicating)	Recombinant novel coronavirus vaccine (Adenovirus type 5 vector)	CanSino Biological Inc./Beijing Institute of Biotechnology	4	X	X	X	X	X	
Viral vector (Non-replicating)	Gam-COVID-Vac	Gamaleya Research Institute; Health Ministry of the Russian Federation	3	X			X	X	X
Viral vector (Non-replicating)	Ad26.COV2.S	Janssen Pharmaceutical	4	X	X	X	X	X	X
Protein subunit	NVX-CoV2373	Novavax	3	X	X	X	X	X	X
RNA based vaccine	mRNA-1273	Moderna + National Institute of Allergy and Infectious Diseases (NIAID)	4	X	X	X	X	X	X
RNA based vaccine	BNT162b2 (Comirnaty)	Pfizer/BioNTech + Fosun Pharma	4	X	X		X	X	X
Protein subunit	CHO Cell	Anhui Zhifei Longcom Biopharmaceutical + Institute of Microbiology, Chinese Academy of Sciences	3						
RNA based vaccine	CVnCoV Vaccine	CureVac AG	3	X	X	X	X	X	X
Inactivated virus	SARS-CoV-2 vaccine (vero cells)	Institute of Medical Biology + Chinese Academy of Medical Sciences	3	X	X		X	X	X
Inactivated virus	QazCovid-in®	Research Institute for Biological Safety Problems, Rep of Kazakhstan	3	X	X		X	X	X
DNA based vaccine	INO-4800	Inovio Pharmaceuticals + International Vaccine Institute + Advaccine (Suzhou) Biopharmaceutical Co., Ltd	2/3	X	X	X	X	X	X
DNA based vaccine	AG0301-COVID19	AnGes + Takara Bio + Osaka University	2/3	X	X		X	X	X
DNA based vaccine	nCov vaccine	Zydus Cadila	3	X	X		X	X	X
Inactivated virus	Covaxin	Bharat Biotech International Limited	3	X	X	X	X	X	X
Inactivated virus	Inactivated SARS-CoV-2 vaccine (Vero cell)	Shenzhen Kangtai Biological Products Co., Ltd.	3		X	X	X	X	X
Protein subunit	EpiVacCorona	Federal Budgetary Research Institution State Research Center of Virology and Biotechnology “Vector”	3	X	X	X	X	X	X

## Data Availability

Not applicable.
